# 6q25.1 (*TAB2*) microdeletion is a risk factor for hypoplastic left heart: a case report that expands the phenotype

**DOI:** 10.1186/s12872-020-01404-5

**Published:** 2020-03-17

**Authors:** Andrew Cheng, Whitney Neufeld-Kaiser, Peter H. Byers, Yajuan J. Liu

**Affiliations:** 1 Department of Cardiology, University of Washington School of Medicine, 1959 NE Pacific Street, Seattle, WA 98195 USA; 2 Department of Pathology, University of Washington School of Medicine, 1959 NE Pacific Street, Room H-478, Seattle, WA 98195-7470 USA; 3 Departments of Pathology and Medicine (Medical Genetics), University of Washington School of Medicine, 1959 NE Pacific Street, Seattle, WA 98195 USA; 4 Departments of Pathology and Laboratory Medicine, University of Washington School of Medicine, 1959 NE Pacific Street, Room H-474B, Seattle, WA 98195-7470 USA

**Keywords:** Hypoplastic left heart syndrome, TAB2 gene deletion, Bicuspid aortic valve

## Abstract

**Introduction:**

Hypoplastic left heart syndrome (HLHS) is a rare but devastating congenital heart defect (CHD) accounting for 25% of all infant deaths due to a CHD. The etiology of HLHS remains elusive, but there is increasing evidence to support a genetic cause for HLHS; in particular, this syndrome is associated with abnormalities in genes involved in cardiac development. Consistent with the involvement of heritable genes in structural heart abnormalities, family members of HLHS patients have a higher incidence of both left- and right-sided valve abnormalities, including bicuspid aortic valve (BAV).

**Case presentation:**

We previously described (Am J Med Genet A 173:1848–1857, 2017) a 4-generation family with a 6q25.1 microdeletion encompassing *TAB2*, a gene known to play an important role in outflow tract and cardiac valve formation during embryonic development. Affected adult family members have short stature, dysmorphic facial features, and multiple valve dysplasia, including BAV. This follow-up report includes previously unpublished details of the cardiac phenotype of affected family members. It also describes a baby recently born into this family who was diagnosed prenatally with short long bones, intrauterine growth restriction (IUGR), and HLHS. He was the second family member to have HLHS; the first died several decades ago. Postnatal genetic testing confirmed the baby had inherited the familial *TAB2* deletion.

**Conclusions:**

Our findings suggest *TAB2* haploinsufficiency is a risk factor for HLHS and expands the phenotypic spectrum of this microdeletion syndrome. Chromosomal single nucleotide polymorphism (SNP) microarray analysis and molecular testing for a *TAB2* loss of function variant should be considered for individuals with HLHS, particularly in those with additional non-cardiac findings such as IUGR, short stature, and/or dysmorphic facial features.

## Introduction

**Fig. 1:**
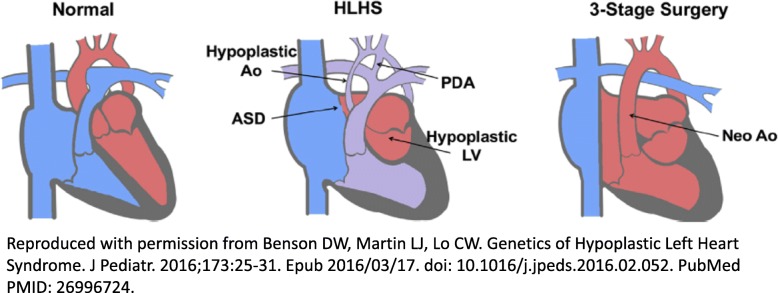
Hypoplastic left heart syndrome and its surgical repair. Legend: HLHS involves a hypoplastic aorta and left ventricle, a large patent ductus arteriosus (PDA), and an atrial septal defect (ASD). This results in a mixture of oxygenated (from PDA flow) and deoxygenated blood flow to the body. A 3-stage surgical repair involves ligation of the PDA, construction of a neo-Aorta, and a baffle in the right atrium that guides deoxygenated blood into the pulmonary circulation. (Reproduced with permission from Benson DW, Martin LJ, Lo CW. [1])

Hypoplastic left heart syndrome (HLHS) is a severe, complex congenital heart defect (CHD) characterized by hypoplasia of the left ventricle and ascending aorta, an atrial septal defect (either large or restrictive), and a patent ductus arteriosus, which provides the only blood flow to the body. It commonly involves atresia or stenosis of the mitral and aortic valves. The prevalence of HLHS is 1.6 per 10,000 live births, and it accounts for 4–8% of all CHD [1]. HLHS is the most severe abnormality in the spectrum of left-sided obstructive CHDs, though it can also be associated with malformation of the tricuspid and pulmonary valves [2]. Although HLHS can be present in a liveborn child, outcomes are universally fatal during infancy without early surgical intervention. Surgical intervention was first implemented in the 1980s, and now involves multiple staged procedures. The end result is that deoxygenated blood is passively directed to the pulmonary circulation via intra-atrial lateral tunnel palliations or more commonly via an extracardiac Fontan circuit (where deoxygenated blood is diverted from the right heart altogether); the right ventricle becomes the systemic ventricle, pumping oxygenated blood through a neo-aorta to the rest of the body (Fig. 1) [1]. Despite surgical advances, HLHS still accounts for 25% of CHD death in infancy, and only 50–70% of affected children live past 5 years of age [2].

The pathogenesis of HLHS is unclear, but there is growing literature supporting a genetic etiology. HLHS is highly heritable, with a 500-fold increased incidence among siblings and a 1000-fold increase if a parent has any form of CHD [3]. Approximately 30% of fetuses with HLHS have genetic syndromes or other extra-cardiac abnormalities [4]. Several syndromes caused by chromosomal abnormalities have been associated with HLHS, including Turner syndrome (monosomy X), Edwards syndrome (trisomy 18), DiGeorge syndrome (deletion of 22q11.21), and Jacobsen syndrome (deletion of 11q) [4–6]. Isolated variants in genes involved in cardiac development have been associated with HLHS (Table 1).

**Table 1:** Genes Associated with Hypoplastic Left Heart Syndrome

Gene	Chromosome	Function
*ERBB4* [7]	2q34	Encodes a tyrosine protein kinase; required for normal cardiac muscle differentiation during embryonic development, and for postnatal cardiomyocyte proliferation.
*HAND1* [8]	5q33.2	Encodes a basic helix-loop-helix transcription factor important in the formation of the right ventricle and aortic arch arteries
*NKX2–*5 [9–11]	5q35.1	Encodes a homeobox-containing transcription factor important in heart formation and development
*NOTCH1* [12–14]	9q34.3	Encodes the Notch 1 protein receptor, which sends signals that are important for normal development of many tissues throughout the body, including the aortic valve
*GJA1* [15]	6q22.31	Encodes a gap junction protein, which play a role in cell-to-cell communication by forming channels, or gap junctions, between cells and are found in many tissues, including the heart
*TBX5* [16]	12q24.1	Encodes T-box protein 5, which plays an important role in the growth and development of the interventricular septum of the heart
*MYH6* [17]	14q11.2	Encodes Myosin-6, found in cardiac muscle cells, where it forms part of a larger protein involved in myocyte contractility
*FOXC2* [18]	16q24.1	Encodes a transcription factor involved in a variety of developmental processes including the cardiovascular system

Haploinsufficiency or loss of function of *TAB2* alone has been shown to be responsible for a multi-system disorder including CHDs. We previously described a 4-generation family (the largest reported to date) with a 6q25.1 microdeletion encompassing *TAB2* (*TGF-beta activated kinase 1/MAP3K7 binding protein 2*) [19]. All affected family members were born with cardiac abnormalities, several with aortic valve malformations, including bicuspid aortic valve (BAV). We now update this family description to include details of the cardiac abnormalities in affected members. We also report the confirmed presence of the *TAB2* deletion in a second child in the family to die in infancy from HLHS. These findings suggest that haploinsufficiency of *TAB2* is a risk factor for HLHS, expanding the phenotype of the previously reported 6q25.1 microdeletion syndrome [19].

## Case presentation

**Fig. 2:**
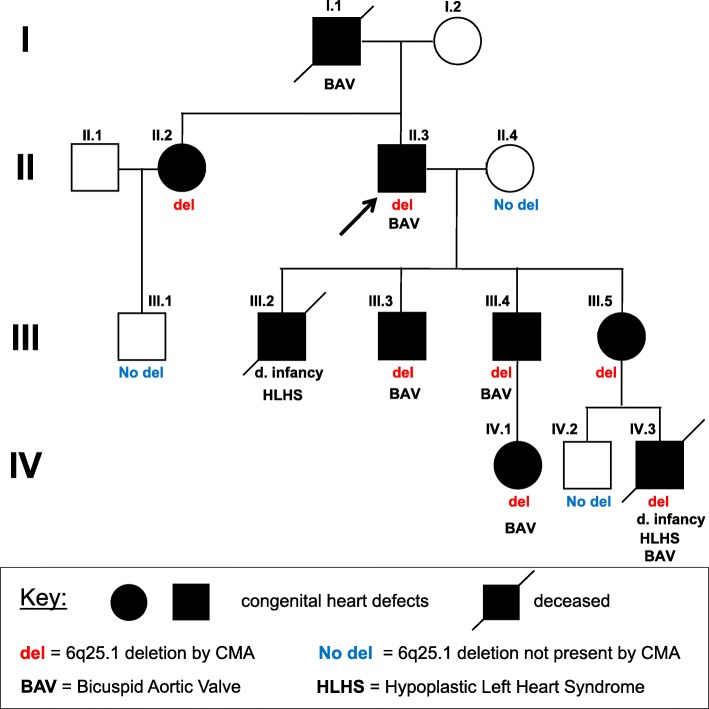
Pedigree of the four-generation family showing **s**egregation of the 6q25.1 deletion with congenital heart defects

**Fig. 3:**
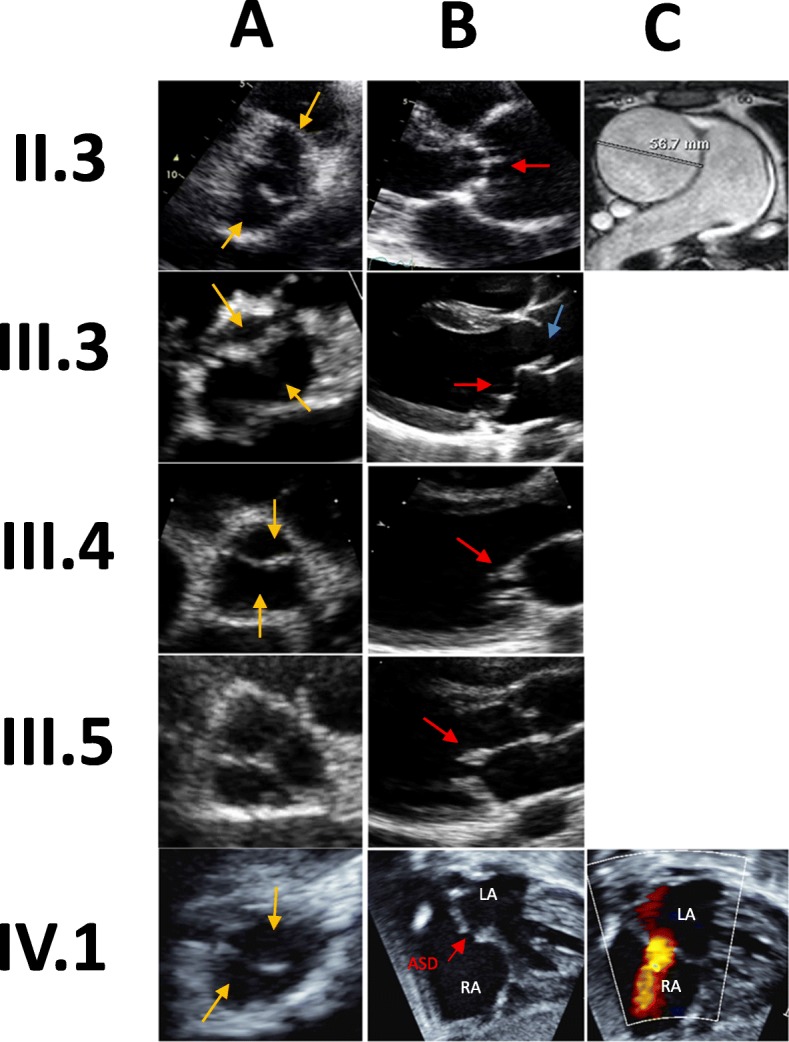
Echocardiogram findings for previously reported family members. Legend: II.3 A: Anterior and posterior aortic leaflets (orange arrows) of a bicuspid aortic valve. II.3B: Calcified aortic valve (red arrow) with a dilated ascending aorta. II.3C: Cardiac MR image showing the ascending aortic aneurysm dilated at 5.8 cm. III.3A: Two leaflets of the bicuspid aortic valve (orange arrows). III.3B: Doming of the aortic valve (blue arrow), consistent with a bicuspid aortic valve, and mitral valve prolapse (red arrow). III.4A: Anterior and posterior leaflets (orange arrows) of a bicuspid aortic valve. III.4B: Thickened mitral valve (red arrow). III.5A: Tri-leaflet aortic valve. III.5B: Thickened mitral valve leaflets (red arrow). IV.1A: Bicuspid aortic valve leaflets (orange arrows). IV.1B-C: Septal defect between the left and right atrium

This report focuses on the second member of the family to die during infancy from complications related to HLHS (VI.3; Fig. 2). Except for this newborn baby (VI.3, Fig. 2) this family’s syndromic features, including their extra-cardiac findings, have been previously described [19]. In this report we highlight their echocardiographic findings. For the cardiovascular manifestations in the family, the proband (II.3) was born with BAV and developed progressive aortic dilation, and he ultimately required aortic valve replacement and aortic root repair (Fig. 3). The proband’s father (I.1) also had BAV, along with mitral valve prolapse and a redundant tricuspid valve; he ultimately died from heart failure. The proband had 4 children (III.2-III.5), all born with congenital valve malformations. The first child (III.2) died within a week of birth. He was the first family member to have HLHS, characterized by a hypoplastic/diminutive left ventricle with a large atrial septal defect, dysplastic aortic valve with aortic stenosis, hypoplastic aorta and aortic arch, aortic coarctation, and a large patent ductus arteriosus. He also had redundant atrioventricular valves.

The second child (III.3) has BAV, bileaflet mitral valve prolapse, and a myxomatous tricuspid valve (Fig. 3). Likewise, the third child (III.4) has BAV and substantial mitral valve thickening (Fig. 3). III.4 has a daughter (IV.1), who was also born with BAV, an atrial septal defect, and a mildly dysplastic pulmonic valve (Fig. 3). Both the fourth child (III.5) and the proband’s sister (II.2) have normal aortic valves but thickened/redundant mitral valve leaflets with mild-to-moderate mitral regurgitation (Fig. 3).

Given the proband’s (II.3) enlarged aorta and bicuspid aortic valve, genetic testing initially focused on genes known to be involved in connective tissue disorders (Marfan, Loeys-Dietz, Ehler-Danlos, Noonan syndromes) and/or BAV (NOTCH1; NKX2–5); no pathogenic variants were identified. Subsequent chromosomal microarray analysis (CMA) performed on the proband (II.3) detected a 1.76 Mb deletion of chromosome 6q24.3-q25.1 ([hg19] chr6:148684028–150,448,233). No other pathogenic copy number variants (CNVs) were present. Testing of the remaining living members of the family showed that the deletion segregates with CHD (Fig. 2). Genetic testing was not possible for III.2, who had died years earlier in the newborn period from complications of HLHS. Thus, while highly probable, it was not definitive that III.2 with HLHS had the familial microdeletion.

**Fig. 4:**
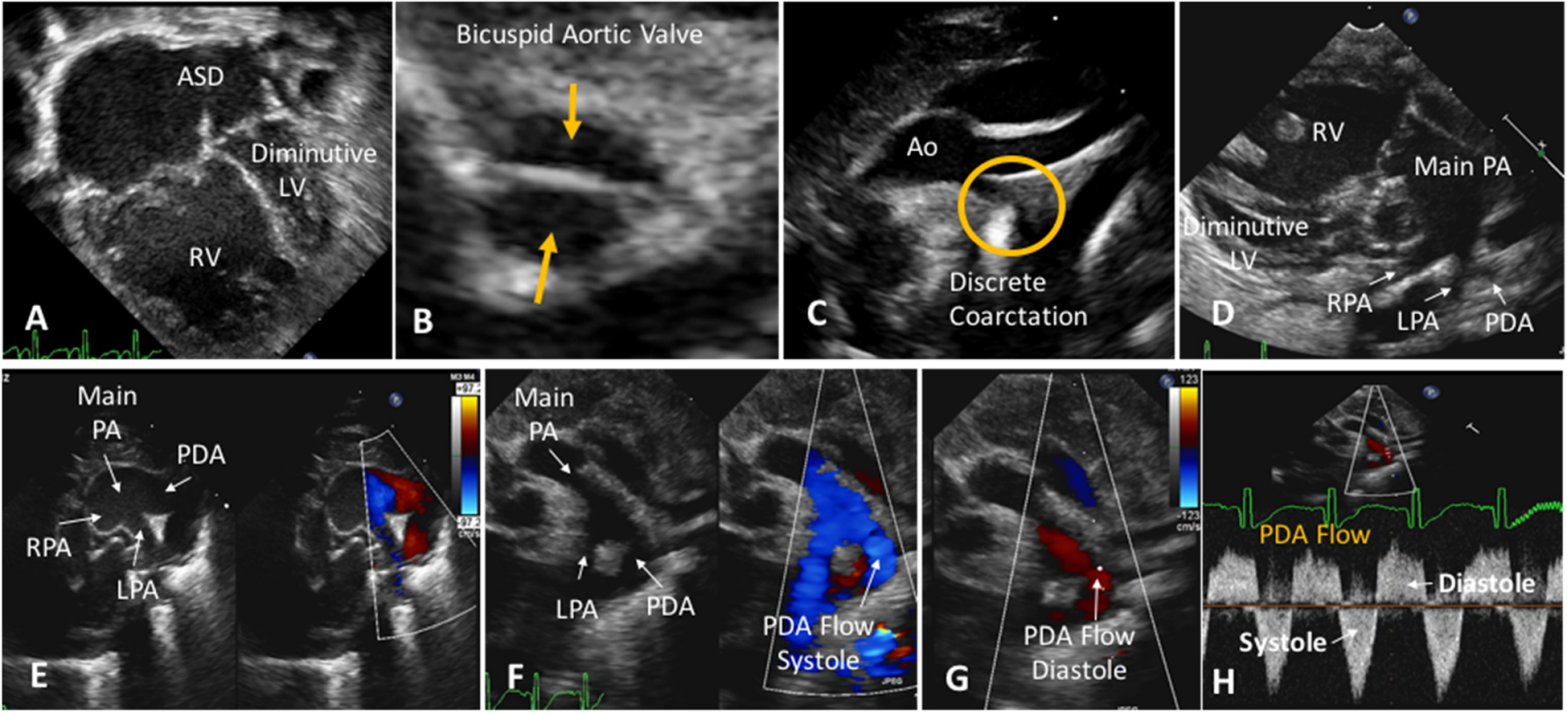
Echocardiogram findings for IV.3. Legend: **a** Large atrial septal defect (ASD) with a diminutive/hypoplastic left ventricle (LV); RV-right ventricle. **b** Orange arrows point to bicuspid aortic valve leaflets. **c** Hypoplastic aorta (Ao) with a discrete coarctation. **d** and **e** Two views of the patent ductus arteriosus (PDA); PA -pulmonary artery; RPA - right pulmonary artery; LPA - left pulmonary artery. **f-g-h** Color and spectral Doppler of the to-and-fro PDA flow from the PA and Ao

However, III.5 recently had a son (IV.3) also born with HLHS. In addition to HLHS, fetal ultrasound revealed short long bones, intrauterine growth restriction, and a horseshoe kidney. The baby was born at 39 weeks. Birth length was 44.5 cm (< 1%), and birth weight was 3.21 kg (39%). Physical exam on day one of life revealed a sacral dimple and syndromic facies with low-set, posteriorly rotated ears. Despite being born at term, the baby had lung hypoplasia and developed severe respiratory distress. Postnatal echocardiogram showed a diminutive, hypoplastic left ventricle with a parachute mitral valve and BAV. He had a hypoplastic aortic arch with a discrete coarctation. He also had an unrestricted atrial septal defect and a large patent duct arteriosus providing systemic blood flow (Fig. 4). His respiratory distress worsened, and he was too unstable for surgical palliation. The baby died 15 days after birth. Postnatal CMA performed on umbilical cord blood, using the Agilent GGXChip + SNP v1.0 4x180K array platform described previously [19], detected the same microdeletion encompassing *TAB2* as seen in the rest of the affected family members.

## Discussion and conclusions

This family’s 6q24.3–25.1 deletion is 1.76 Mb and spans 21 genes (Supplemental Figure 1) [19]. There are multiple lines of evidence implicating *TAB2* as the causal gene for structural CHD in this region [19–22], though we cannot definitely exclude involvement of the 20 other genes in this family’s structural heart disease. *TAB2* is heavily expressed in the endocardial cushion and plays an important role in outflow tract and valvular formation during human embryonic development. Titrated knockdown of TAB2 in embryonic zebrafish showed dose-sensitive defects in cardiac development [20]. *TAB2* was shown to be the only gene within the smallest overlapping region among patients with a 6q25.1 microdeletion and CHD [19], and a balanced translocation that disrupted *TAB2* was shown to segregate with familial CHD [20]. Ackerman et al. recently reported a child born with a similar CHD presentation with a sporadic *TAB2* nonsense variant (c.1491 T > A; p.Y497X) [21]. *TAB2* microdeletions have also been associated with more complex CHD, including tetralogy of Fallot [22]. Our report is the first associating *TAB2* haploinsufficiency with HLHS.

Hitz [23] and Carey [24] hypothesize that up to 10% of HLHS is related to chromosomal microdeletions or duplications. In this family with a known chromosomal deletion, two members in differing generations died of HLHS, one of which was verified to have the *TAB2* microdeletion. It is unlikely that this is coincidental and unrelated to the deleted gene known to affect cardiac development. Generational skips in phenotype could be related to an autosomal recessive inheritance pattern, but given the rarity of HLHS, the odds of autosomal recessive inheritance are extremely low. The family’s phenotypic and genotypic findings suggest that haploinsufficiency of *TAB2* is a risk factor for HLHS. As we collect genetic data on cohorts of individuals with HLHS, it will be worthwhile to see if a 6q25.1 deletion/*TAB2* abnormality is more pervasive in this population.

In our 4-generation family, BAV is widely prevalent. A genetic relationship between HLHS and BAV has long been speculated [25]. Approximately 10% of relatives of infants with HLHS have BAV, whereas BAV is present in only 1–2% of the general population [26, 27] . Hinton et al. reported a set of monozygotic twins, one with BAV and the other with HLHS [3]. Pathogenic variants in other genes, such as *NOTCH1*, cause a spectrum of aortic valve abnormalities, including both BAV [13] and HLHS [28]. Based on observation alone, we cannot definitively prove that BAV and HLHS co-segregate within the family through a common genetic defect. However, like *NOTCH1, TAB2* is important in embryonic cardiac development [20]. *TAB2* deletions and loss-of-function variants have been shown to cause a variety of left-sided obstructive lesions, so it would not be surprising that BAV and HLHS are both within the phenotypic spectrum of *TAB2* haploinsufficiency.

This case report also underscores the complexity of genotype-phenotype predictions. Even within a single family with the identical 6q25.1 microdeletion, there is great variability in the spectrum of CHD, from simple valvular defects to HLHS. Variable expressivity, genetic heterogeneity, and reduced penetrance have been proposed as possible factors contributing to genotype-phenotype differences in CHD [1]. *TAB2* appears to be a risk factor for HLHS, but there may be other genetic modifiers and environmental factors critical to the development of this congenital abnormality. Recently, a study using 8 mouse lines with HLHS highlights the genetic heterogeneity of HLHS. Exome sequencing revealed 330 coding or splicing mutations, none which were shared among the mouse lines. In addition, 5 mouse lines had pathogenic variants in 2 or more genes in analogous human chromosomal regions previously associated with HLHS or LV outflow tract obstruction [29]. This discovery favors a multigenic etiology for HLHS. Perhaps the two family members affected with HLHS (III.2 and IV.3) had additional genetic variants predisposing them to more complex CHD.

Although we cannot yet predict who with a *TAB2* deletion will have HLHS, our observation that *TAB2* haploinsufficiency is associated with HLHS is an important step in further elucidating the genetic underpinnings of this complex congenital heart disease. Thus far, only a handful of gene abnormalities have been linked with this CHD. Given the universal mortality associated with HLHS without early palliation, and the newly recognized association with the 6q25.1 microdeletion, we recommend a fetal echocardiogram in all women carrying an at-risk fetus. Pre-conception genetic counseling is recommended for affected individuals, even those with only a mild phenotype. Furthermore, testing for abnormalities in *TAB2* should be considered in patients with HLHS with any non-cardiac abnormalities, including prenatal growth restriction, short stature, and/or dysmorphic facial features. Given the likely genetic heterogeneity of HLHS, chromosomal microarray analysis to evaluate for microdeletions, reflexing to molecular testing for *TAB2* loss of function variants should be standard in the genetic work-up of these patients.

## Supplementary information

**Additional file 1: Supplemental Figure 1.** Chromosomal microarray analysis (CMA) results of VI.3 with HLHS using Agilent GGXChip + SNP v1.0 4x180K array. The deletion detected in VI.3 was a 1.76 Mb deletion of chromosome 6q24.3-q25.1 ([hg19] chr6:148684028–150,448,233) and inherited from his mother (III.5). X axis: log2 Ratio. Y axis: genomic location. Non-mosaic one-copy deletions have log2R = − 1. UCSC genes in the deleted region.

## Data Availability

Chromosomal microarray analysis data for this study is available upon request from the corresponding author.

## Abbreviations

BAV: Bicuspid aortic valve
CHD: Congenital heart defect
CMA: Chromosomal microarray analysis
HLHS: Hypoplastic left heart syndrome
TAB2: TGF-beta activated kinase 1/MAP3K7 binding protein 2
