# Supplementary material for: 6q25.1 (TAB2) microdeletion is a risk factor for hypoplastic left heart: a case report that expands the phenotype
Source: BMC Cardiovasc Disord. 2020 Mar 17;20:137. doi: 10.1186/s12872-020-01404-5 (PMC7077097; doi:10.1186/s12872-020-01404-5)
Supplement: Supplementary file 1 — Additional file 1: Supplemental Figure 1. Chromosomal microarray analysis (CMA) results of VI.3 with HLHS using Agilent GGXChip + SNP v1.0 4x180K array. The deletion detected in VI.3 was a 1.76 Mb deletion of chromosome 6q24.3-q25.1 ([hg19] chr6:148684028–150,448,233) and inherited from his mother (III.5). X axis: log2 Ratio. Y axis: genomic location. Non-mosaic one-copy deletions have log2R = − 1. UCSC genes in the deleted region. [file 12872_2020_1404_MOESM1_ESM.pptx]

## Slide 1
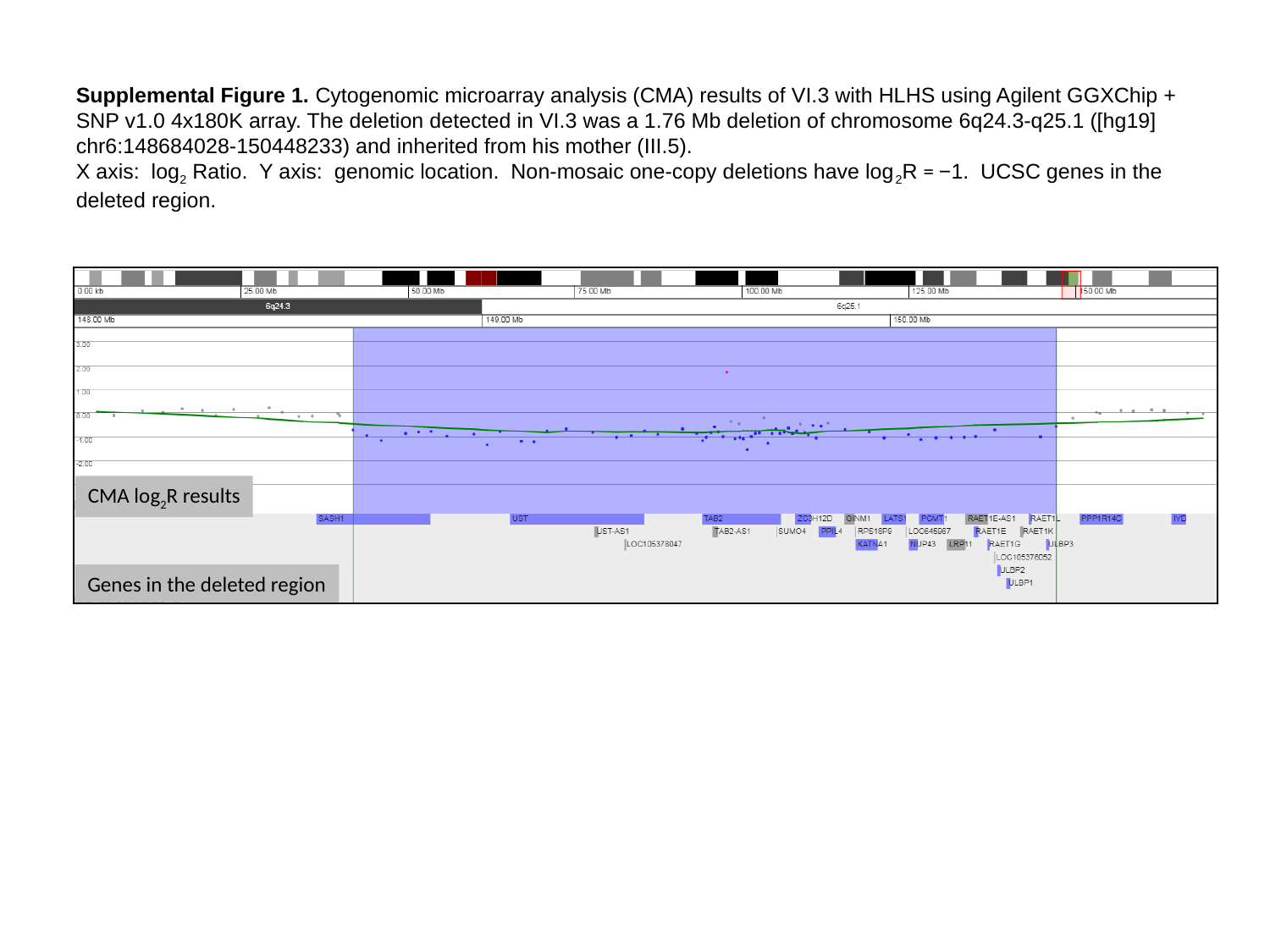

Supplemental Figure 1. Cytogenomic microarray analysis (CMA) results of VI.3 with HLHS using Agilent GGXChip + SNP v1.0 4x180K array. The deletion detected in VI.3 was a 1.76 Mb deletion of chromosome 6q24.3-q25.1 ([hg19] chr6:148684028-150448233) and inherited from his mother (III.5).
X axis: log2 Ratio. Y axis: genomic location. Non-mosaic one-copy deletions have log2R = −1. UCSC genes in the deleted region.
CMA log2R results
Genes in the deleted region
